# Bile Acids and Type 2 Diabetes: Roles in Glucose Homeostasis and Therapeutic Opportunities

**DOI:** 10.3390/metabo15060401

**Published:** 2025-06-13

**Authors:** Yiting Lin, Chunyan Hu, Shuangyuan Wang, Hong Lin

**Affiliations:** 1 Department of Endocrine and Metabolic Diseases, Shanghai Institute of Endocrine and Metabolic Diseases, Ruijin Hospital, Shanghai Jiao Tong University School of Medicine, Shanghai 200025, China; linyt0202@sjtu.edu.cn (Y.L.); hcy01j96@rjh.com.cn (C.H.); 2 Shanghai National Clinical Research Center for Endocrine and Metabolic Diseases, Key Laboratory for Endocrine and Metabolic Diseases of the National Health Commission of the PR China, Shanghai National Center for Translational Medicine, Ruijin Hospital, Shanghai Jiao Tong University School of Medicine, Shanghai 200025, China

**Keywords:** bile acids, type 2 diabetes, glucose homeostasis, mass spectrometer

## Abstract

Background: Type 2 diabetes mellitus (T2DM), characterized by impaired glucose homeostasis, represents a significant threat to public health. Bile acids (BAs), as key metabolic regulators, play an essential role in glucose metabolism. Recent advances in high-resolution metabolomics have revealed that various BA species are closely linked to T2DM pathogenesis and play a critical role in maintaining glucose homeostasis. Understanding the underlying mechanisms by which BAs modulate glucose metabolism provides valuable insights for the prevention and treatment of T2DM. Methods/Results: This review describes the roles of diverse BA species in regulating glucose metabolism and comprehensively summarizes the relationship of unconjugated and conjugated BAs with T2DM in population studies. Furthermore, we discuss BA-targeted therapeutic approaches for T2DM, highlighting the urgent need for developing tissue-restricted modulators of BA receptors and advancing the clinical translation of novel beneficial BAs. Conclusion: Deeply understanding the role of BAs played in the pathogenesis and progression of T2DM will facilitate the development of potential therapeutic agents.

## 1. Introduction

Type 2 diabetes (T2DM) is a major noncommunicable chronic disease and a significant public health concern. Epidemiological data estimate that approximately 529 million individuals worldwide are living with diabetes across all age groups. The age-standardized prevalence has increased from 3.2% in 1990 to 6.1% and is expected to reach 9.8% (1.31 billion people) in 2050 [1,2]. Notably, T2DM accounts for nearly 96% of all diabetes cases [1]. This heterogeneous metabolic disease is characterized by impaired glucose homeostasis, leading to chronic hyperglycemia. Two fundamental pathophysiological mechanisms of T2DM are insulin insufficiency due to pancreatic β cell dysfunction and insulin resistance (IR) in peripheral tissues. Emerging evidence highlights the critical role of metabolites in the onset and progression of T2DM [3,4,5,6]. Among these, bile acids (BAs) have emerged as important players. Understanding the distinct ways in which BAs influence glucose metabolism could deepen our insights into T2DM pathogenesis and inform the development of more effective prevention and treatment strategies.

BAs are cholesterol-derived molecules synthesized in hepatocytes, serving as amphipathic surfactants and systemic endocrine molecules. BAs, traditionally known for their role in lipid emulsification and intestinal absorption [7], have been recognized as critical regulatory factors in metabolic processes, particularly in glucose metabolism and the secretion of glucoregulatory hormones [8]. Growing evidence from both observational and experimental studies links altered BA metabolism to the onset and progression of T2DM [9,10]. The nuclear farnesoid X receptor (FXR) and the membrane-bound Takeda G protein-coupled receptor 5 (TGR5) are two well-characterized BA receptors that play a vital role in regulating glucose metabolism, including the inhibition of gluconeogenesis, increase in insulin sensitivity, stimulation of glucagon-like peptide-1 (GLP-1) and insulin secretion, suppression of inflammatory responses, and regulation of gut microbial homeostasis [11,12,13]. It is worth noting that varied BA species exhibit distinct physicochemical properties, resulting in receptor-independent effects on downstream metabolic pathways [14,15]. Therefore, elucidating the BA-mediated mechanisms in glucose metabolism and their relationship with T2DM is essential for developing novel therapeutic strategies.

Metabolomics, an emerging omics field, serves as a powerful tool for both qualitative and quantitative analysis of metabolites [16,17]. This approach enables precise detection of known and novel small-molecule metabolites. BAs, a structurally diverse metabolite family characterized by extensive modifications, are usually analyzed by non-targeted or targeted methodologies using a liquid chromatograph mass spectrometer (LC-MS) platform [18,19]. A recent breakthrough study integrated 1.2 billion publicly available tandem mass spectrometry (MS/MS) spectra to construct the most comprehensive BA profile to date [20]. These technological improvements have profoundly advanced our understanding of specific BAs involved in regulating glucose metabolism.

In this review, we aim to provide a comprehensive overview of the mechanisms by which diverse BAs regulate glucose metabolism and discuss the application of advanced metabolomics techniques in elucidating BA-T2DM associations in observational studies. Furthermore, we introduce BA-targeted therapeutic strategies for T2DM, aiming to reveal their potential value for clinical application.

## 2. Bile Acids Synthesis and Enterohepatic Circulation

**Figure 1:**
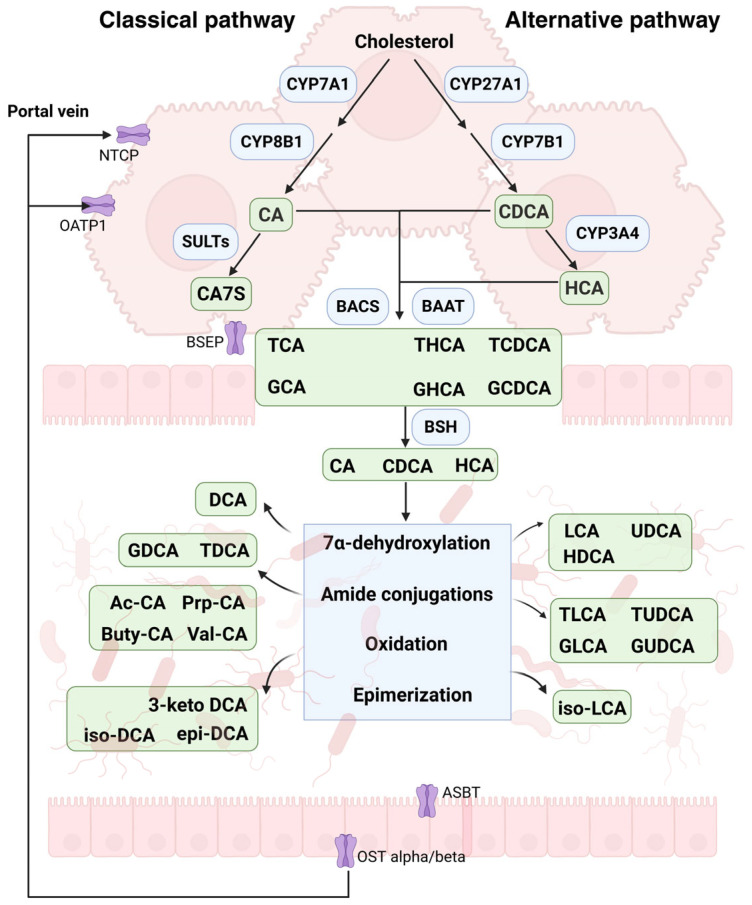
Bile acid synthesis and enterohepatic circulation. The figure depicts the process of bile acid synthesis through classical and alternative pathways and demonstrates enterohepatic circulation. This figure was created in Biorender. Yiting Lin. (2025) https://BioRender.com/.

Primary unconjugated BAs, including cholic acid (CA) and chenodeoxycholic acid (CDCA), are synthesized from cholesterol in the liver via two parallel metabolic pathways (Figure 1). The classical pathway, which accounts for approximately 75% of BA synthesis, is initiated by the rate-limiting enzyme cholesterol 7α-hydroxylase (CYP7A1). CYP7A1 serves as the primary regulator of BA biosynthesis through catalyzing the conversion of cholesterol into 7α-hydroxycholesterol [21]. Subsequently, sterol 12α-hydroxylase (CYP8B1) facilitates the formation of CA and CDCA [22], playing a pivotal role in determining their ratio and regulating their overall production [9,23]. The alternative pathway involves sterol 27-hydroxylase (CYP27A1) catalyzing the conversion of cholesterol to 27-hydroxycholesterol, followed by further transformation into CDCA by oxysterol 7α-hydroxylase (CYP7B1) [24]. Although 6α-hydroxylation of BAs in humans has not been extensively studied, it is known that hyocholic acid (HCA) is produced in hepatocytes from CDCA via human hepatic CYP3A4 [25].

In hepatocytes, most BAs are conjugated with glycine and taurine to form primary conjugated BAs. These processes are driven by the enzymatic activity of bile acid: CoA synthetase (BACS) and bile acid–CoA: amino acid N-acyltransferase (BAAT) [24]. The primary conjugated BAs are transferred into the biliary system through the bile salt export pump (BSEP) and stored in the gallbladder [24]. Upon food intake, gallbladder contraction releases them into the intestinal lumen. Initially, primary conjugated BAs are deconjugated into unconjugated BAs by the activity of bile salt hydrolase (BSH) [26]. Thereafter, secondary unconjugated BA, including deoxycholic acid (DCA), iso-DCA, epi-DCA, 3-keto DCA, lithocholic acid (LCA), iso-LCA, ursodeoxycholic acid (UDCA), and so on, may be generated via dehydroxylation, oxidation, and epimerization. Among these processes, 7α-dehydroxylation is considered the most critical reaction. Notably, 3-O-acyl-CA is a novel class of secondary BAs synthesized by *Christensenella minuta* via acylation at the 3-hydroxy ends of CA, encompassing 3-acetyl cholic acid (Ac-CA), 3-propionyl cholic acid (Prp-CA), 3-butyryl cholic acid (Buty-CA), and 3-valeryl cholic acid (Val-CA) [27]. Secondary conjugated BAs will be generated when secondary unconjugated BAs conjugate with glycine or taurine. Most unconjugated BAs are reabsorbed into enterocytes via the sodium-dependent bile acid transporter (ASBT) in the distal ileum. Transporting proteins, including organic solute transporter alpha and beta (OST alpha/beta), ileum bile acid binding protein (IBABP), and sodium-dependent taurocholate cotransporting polypeptide (NTCP), facilitate their entry into the portal vein, where they are transported back to the liver.

The process of BA biosynthesis, transport, and metabolism is referred to as enterohepatic circulation. Optimally, approximately 3 g (around 90–95%) of BAs are recycled and absorbed between the intestines and the liver in roughly eight cycles daily, while only about 0.2–0.6 g per day are synthesized from de novo bile acids to sustain a stable bile acid pool. The remaining bile acids, which are not reintroduced into the hepatocyte (approximately 5%), are excreted in feces or urine.

**Figure 2:**
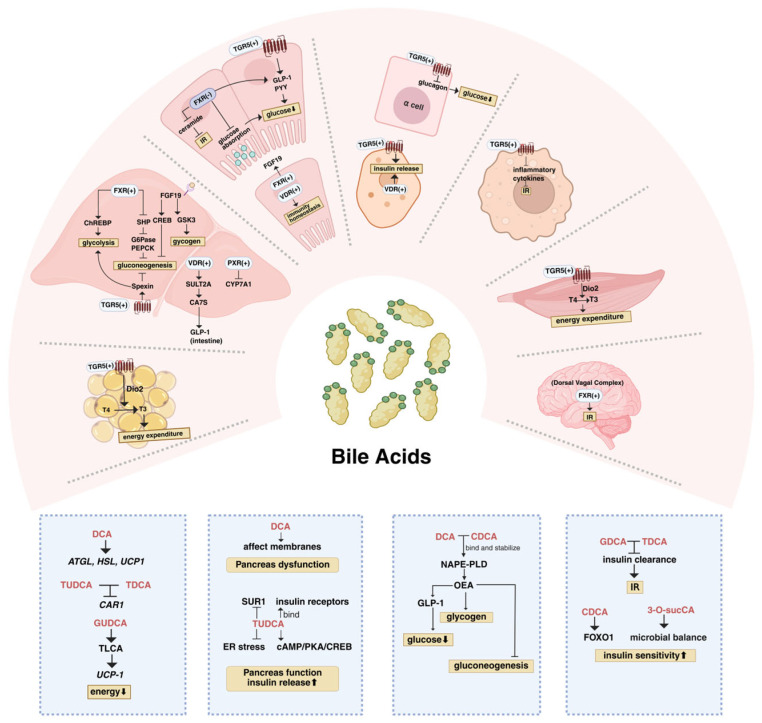
Mechanisms of bile acid regulating glucose metabolism. The figure depicts classical receptor-mediated mechanisms function across multiple tissues (top panel) and non-receptor-mediated mechanisms (bottom panel). Non-receptor-mediated mechanisms are categorized in blue boxes according to functional roles: (1) energy absorption and expenditure, (2) pancreatic function and insulin release, (3) glycogen synthesis and gluconeogenesis, and (4) insulin resistance and sensitivity. (+), activation; (−), inhibition. This figure was created in Biorender. Yiting Lin. (2025) https://BioRender.com/.

BAs modulate glucose metabolism primarily through classical receptor-mediated mechanisms, and emerging evidence has suggested that non-receptor-mediated mechanisms may contribute to it, which enriches our understanding of the BA metabolic regulatory network. This section will detail receptor-mediated mechanisms of BA action on glycemic control and examine non-receptor-mediated regulatory pathways (Figure 2).

## 3. Mechanisms of BAs in the Regulation of Glucose Homeostasis

### 3.1. Receptor-Mediated Mechanisms

#### 3.1.1. FXR

FXR is the first discovered BA receptor, predominantly expressed in the liver, intestine, and kidney [28]. Both activation and inhibition of FXR play crucial roles in glucose metabolism regulation. Hepatic FXR activation upregulates the expression of the small heterodimer partner (SHP), an inhibitory nuclear receptor [29,30]. SHP suppresses the activity of various transcription factors and downregulates key gluconeogenic genes, including glucose-6-phosphatase (G6Pase) and phosphoenolpyruvate carboxylase kinase (PEPCK) [31,32,33,34]. Additionally, FXR can directly interact with carbohydrate response element-binding protein (ChREBP) to regulate glycolysis [35,36]. In the intestine, FXR activation induces the release of the peptide hormone fibroblast growth factor 19 (FGF19) [37]. They can reduce hepatic gluconeogenesis by promoting the dephosphorylation of the gluconeogenic transcription factor CREB [38]. The ERK-GSK3α/β phosphorylation cascade can be initiated by FGF19, enhancing hepatic glycogen synthesis [39] and contributing to weight reduction [40]. Suppression of intestinal FXR can diminish ceramide synthesis in the intestine, hence alleviating endoplasmic reticulum (ER) stress and the secretion of proinflammatory cytokines in the liver, which improves IR [41]. FXR inhibition also elevates glucose phosphorylation levels in intestinal epithelial cells and postpones intestinal glucose absorption to regulate blood glucose levels [42]. Moreover, inhibition of intestinal FXR promotes the transcription and synthesis of proglucagon, thereby enhancing GLP-1 secretion [43]. Whether FXR activation or inhibition predominates in maintaining glucose homeostasis is highly dependent on tissue specificity and its net effect on the subject [44]

Diverse BAs exert distinct effects on FXR activation [45]. CDCA is the most potent natural agonist [46], whereas DCA, LCA, and CA act as partial agonists. In contrast, UDCA, glycoursodeoxycholic acid (GUDCA), tauroursodeoxycholic acid (TUDCA), and HCA species (hyodeoxycholic acid (HDCA), glycohyocholic acid (GHCA), glycohyodeoxycholic acid (GHDCA), taurohyocholic acid (THCA), and taurohyodeoxycholic acid (THDCA)) function as FXR inhibitors [47]. Current research also showed that 3-O-acyl-CA engaged in metabolic pathways associated with energy metabolism and insulin sensitivity by blocking intestinal FXR [27]. It is worth noting that the previously overlooked taurochenodeoxycholic acid (TCDCA)-FXR axis has recently garnered growing attention. It has been shown that a high-fat diet (HFD) alters the microbial composition in the upper small intestine, leading to increased levels of TCDCA in the intestine, plasma, and dorsal vagal complex (DVC) [48]. Experimental evidence confirms that increased TCDCA levels activate DVC FXR, which impairs insulin function and subsequently reduces hepatic glucose production. Conversely, direct inhibition of FXR in the DVC restored the ability of insulin to lower hepatic glucose production in HFD rats with hepatic IR [48]. Moreover, the impact of the TCDCA-FXR axis on insulin function may be linked to the modulation of glutamatergic and GABAergic neurons in the nucleus tractus solitarius of the DVC [48,49]. These findings highlighted the crucial role of the TCDCA-FXR axis in glucose homeostasis.

#### 3.1.2. TGR5

TGR5, a BA-specific G-protein-coupled receptor, can activate multiple intracellular signaling pathways in response to BA binding [50]. It is extensively expressed in various human tissues, including pancreatic β-cells, endocrine L cells in the small intestine, thyroid, brown adipose tissue (BAT), cardiomyocytes, macrophages, and hepatic sinusoidal endothelial cells [30,45,51]. TGR5 plays a crucial role in glucose homeostasis, with its activation mechanisms exhibiting tissue-specific variability. Recent investigations have shown that TGR5 activation upregulated the hepatic expression of the novel neuropeptide Spexin, which enhances glycolysis and suppresses gluconeogenesis [52,53]. These effects were strongly associated with the AC/cAMP/PKA and MAPK signaling pathways through the activated TGR5 [54]. In the intestine, TGR5 activation enhances GLP-1 synthesis and secretion via the cAMP/PKA signaling pathway in endocrine L cells and promotes the release of peptide tyrosine tyrosine (PYY) to reduce appetite [55,56,57]. In the pancreas, BA-induced TGR5 activation stimulates insulin release from β-cells through AC/cAMP/PKA or cAMP/EPAC/PLC pathways while inhibiting glucagon secretion from α-cells [58,59,60]. Additionally, TGR5 activation facilitates the conversion of thyroxine (T4) into triiodothyronine (T3) via type 2 iodothyronine deiodinase in the BAT and muscle [47], thereby enhancing thermogenesis and energy expenditure [61]. In immune cells, TGR5 activation suppresses the production of inflammatory cytokines, thereby improving insulin sensitivity [62].

DCA and LCA are the most effective activators of the TGR5 receptor [63]. CA-7-sulfate (CA7S) [64] was found to have an anti-diabetic effect by enhancing TGR5 expression [65]. Recent intriguing findings demonstrated that HCA species function as dose-dependent agonists of the TGR5. Unlike other bile acid species, which fail to stimulate GLP-1 secretion at higher concentrations (e.g., 50 μM), HCA species retain their efficacy [66,67], suggesting their potential utility at elevated doses for maintaining glucose homeostasis and offering promising therapeutic prospects [67].

#### 3.1.3. Other Receptors

The vitamin D receptor (VDR), a nuclear receptor typically activated by its canonical ligand, 1α,25-dihydroxyvitamin D_3_, regulates diverse physiological processes including immunity and metabolism [68]. While VD is the primary activator, studies show that LCA can also act as a VDR agonist, exhibiting lower potency and requiring high doses to activate VDR in vivo significantly [69]. A recent study in mice undergoing bariatric surgery revealed that LCA can be selectively transported from the intestine to the liver, where it activates hepatic VDR. This activation induces the expression of BA sulfotransferase (SULT2A), leading to the production of the antidiabetic molecule CA7S [70]. This finding suggested that the LCA-VDR-SULT2A-CA7S-GLP-1 pathway may contribute to blood glucose regulation after bariatric surgery in humans [70]. Given that current research on the impact of LCA-VDR signaling on glucose homeostasis is limited, more precise mechanisms remain unclear. Existing evidence suggests that VDR signaling impacts glucose homeostasis through multiple mechanisms: suppressing hepatic glucose production and IR [71], regulating RORγ^+^ Treg cells to maintain colonic Treg cell homeostasis [72], supporting insulin synthesis and secretion while enhancing PYY transcription in the pancreas [73,74], and modulating skeletal muscle insulin sensitivity and glucose tolerance [75].

Notably, the pregnane X receptor (PXR) and constitutive androstane receptor (CAR) are two additional nuclear receptors for BAs. LCA can directly activate PXR and indirectly active CAR to maintain BA homeostasis by inhibiting CYP7A1 via the FGF19 pathway [76].

### 3.2. Non-Receptors-Mediated Mechanism

Mechanisms underlying BA regulation of glucose homeostasis are intricate. Current evidence also supports that diverse BAs can modulate glucose metabolism via multiple mechanisms beyond their effects through the classical receptor pathway. DCA and CDCA have been shown to enhance the activity of N-acyl phosphatidylethanolamine phospholipase D (NAPE-PLD) by directly binding and stabilizing the enzyme [77]. NAPE-PLD is an essential enzyme found in the brain and intestine, with oleoylethanolamide (OEA) as its catalytic product [78]. OEA not only stimulates the release of GLP-1 [79] but also augments glycogen production and suppresses hepatic gluconeogenesis via the LKB1/AMPK signaling pathway [80]. Pancreatic β-cell function appears to be influenced by BAs directly or indirectly. As a highly hydrophobic BA [81], DCA can be incorporated into the plasma membrane and mitochondrial inner membrane [82,83], triggering inflammation, oxidative stress, and pancreatic β-cell dysfunction [84,85]. TUDCA, as a chemical chaperone, may improve pancreatic islet function and enhance insulin secretion by the following mechanisms: it can alleviate ER stress; binds with insulin receptors to activate the AKT pathway [86]; blocks sulfonylurea receptor 1 (SUR1), a subunit of the ATP-sensitive potassium channel, to reduce K+ efflux and elevate cytoplasmic Ca^2+^ concentration [62]; activates the cAMP/PKA/CREB signaling pathway under high-glucose conditions to stimulate insulin release [87]; and normalizes insulin secretion [88] through partially inhibiting the expression of glutamate dehydrogenase (GDH), a key component of insulin secretion amplification [88,89]. With regard to insulin sensitivity, CDCA has been hypothesized to improve skeletal muscle insulin sensitivity through mechanisms independent of classical BA receptors, potentially by modulating insulin signaling via enhanced forkhead box O1 (FOXO1) activity [90]. 3-O-sucCA can enhance a microbial balance that bolsters intestinal barrier integrity and reduces inflammation to augment insulin sensitivity and energy expenditure indirectly [91]. Moreover, elevated serum levels of glycodeoxycholic acid (GDCA) and taurodeoxycholic acid (TDCA) were strongly associated with reduced insulin clearance, potentially exacerbating insulin resistance [92]. It was worth noting that concentration changes of specific BAs may influence the equilibrium levels of other BAs. A recent study found that TDCA functions as an endogenous inhibitor of LCA to improve glucose metabolism by suppressing the expression of the bacterial bile acid-inducible (*bai*) operon without affecting bacterial growth [93]. Another study suggested that elevated GUDCA levels in mice lead to an increase in taurolithocholic acid (TLCA) levels and an enhancement in the abundance of *Bacteroides vulgatus* [94]. Together, these factors activate TGR5 and upregulate uncoupling protein 1 (UCP-1), resulting in increased thermogenesis in white adipose tissue and improved glucose metabolism [94]. DCA can also enhance lipolysis and thermogenesis by upregulating the expression of *ATGL*, *HSL*, and *UCP1* in BAT, thereby preventing HFD-induced obesity and improving glucose tolerance [95]. Additionally, TUDCA and TDCA were reported to suppress energy absorption by remarkably downregulating the expression of intestinal carbonic anhydrase 1 (CAR1), a marker for absorptive intestinal epithelial cells and a possible obesity target [96]. However, the precise mechanisms by which TUDCA and TDCA regulate *CAR1* expression remain to be elucidated.

## 4. BA Profile Alterations in T2DM Pathogenesis

Extensive clinical investigations utilizing MS platforms have systematically characterized the associations between circulating BA profile alterations and T2DM development. These studies have revealed distinct patterns of BA compositional changes in different disease stages and the potential predictive value of BA signatures for T2DM risk. Notably, current evidence suggests that most unconjugated BAs are positively associated with improved metabolic health. In contrast, the majority of conjugated BAs appear to be linked to metabolic dysfunction, except for conjugated HCA. These opposing effects on diabetes risk will be detailed in this section [97]. Table 1 provides a comprehensive summary of key observational studies, including their study designs and major findings, and Table 2 systematically summarizes the current evidence on BA-T2DM epidemiological associations and their potential mechanisms.

### 4.1. Unconjugated BAs

Unconjugated BAs, including CA, DCA, CDCA, LCA, and UDCA, have been widely investigated in several human studies to explore their concentration alterations during T2DM or to evaluate their potential role in predicting T2DM. A large prospective nested case–control study involving 3414 normoglycemic Chinese individuals from a nationwide cohort of the China Cardiometabolic Disease and Cancer Cohort (4C cohort) with a median follow-up of 3.03 years investigated the association between 23 BA species and T2DM risk using UPLC-MS/MS [97]. It firstly suggested that all unconjugated primary BAs (CA and CDCA) and DCA were associated with a decreased risk of T2DM [97]. However, the protective role of DCA remains controversial. Elevated serum levels of DCA have been reported to be associated with the increased risk of T2DM in the Finnish Diabetes Prevention Study (DPS) with a 15-year follow-up [99] and a Swedish research study comprising four high-quality prospective cohorts (Swedish meta-analysis study) among mixed populations with normal glucose regulation (NGR) and impaired fasting glucose (IFG) [98]. Moreover, the same findings were also reported in overweight or obese black women with NGR during a 13-year follow-up [101]. Current cross-sectional study using UPLC-MS/MS to quantify 28 plasma and fecal bile acids in 200 paired individuals (100 treatment-naive T2DM patients and 100 BMI-matched NGR controls) found that plasma levels of DCA, iso-DCA, and 12epi-DCA were significantly elevated in T2DM and that both plasma and fecal DCA were associated with HOMA-IR, fasting blood glucose (FBG), glycated hemoglobin (HbA1c), and insulin, highlighting a strong link between DCA and impaired glucose metabolism [102]. It was worth noting that conflicting findings in longitudinal studies may stem from several factors. Variations in sample size, participant ethnicity, BA quantification technology, follow-up duration, and baseline metabolic status may influence results. Biologically, genetic variants associated with circulating DCA levels frequently show population-specific differences in effect allele frequencies, which may lead to inherent differences in baseline plasma DCA concentrations [9,106]. In addition, metabolic conditions such as obesity or IFG may alter the composition and function of gut microbiota involved in DCA production, contributing to inter-individual variability at baseline [76]. These differences may accumulate over time, influencing the results and interpretation of longitudinal studies.

UDCA has been extensively researched for its therapeutic efficacy in the treatment of cholestatic liver disorders. Multiple investigations have indicated that UDCA plays a significant role in improving glucose metabolism [107,108]. Longitudinal cohort studies suggested that participants with lower baseline UDCA levels have a higher risk of developing T2DM [101]. Recent clinical trials have reported a notable decrease in FBG and HbA1c levels in the UDCA treatment group [109].

Elevated plasma LCA levels were found in T2DM compared to individuals with NGR, and the levels reversed following antidiabetic treatment in a clinical study using LC-MS/MS as a measurement tool. More recently, a case–control study involving 30 T2DM and 50 healthy subjects measured BA levels using UPLC/MS-MS and reported significantly elevated serum LCA levels in T2DM compared to healthy controls, further supporting previous findings [94]. However, prospective longitudinal cohort studies have not demonstrated sufficient evidence to support LCA as a predictive biomarker for T2DM risks.

HCA species, accounting for only about 3% of the plasma of humans and rats (compared to more than 75% in pig plasma) [104], remained uncharacterized until advancements in MS sensitivity enabled animal studies and population-based observational studies to investigate their associations with metabolic diseases. In 2020, Talavera et al. conducted a 5-year prospective cohort study within the IT-DIAB cohort study first to explore the relationship between circulating BAs and glycemic parameters in 205 individuals with IFG using LC-MS/MS [110]. The findings of this study demonstrated that HCA exhibited a negative correlation with HOMA-IR, emphasizing its potential function in glucose homeostasis [110]. These observations were supported by subsequent research encompassing five human studies [104]. Two cross-sectional studies demonstrated that HCA and HDCA in serum and feces had significant inverse associations with fasting or post-load blood glucose levels, and improvement of these two clinical glucose parameters was observed in T2DM after Roux-en-Y gastric bypass surgery [104]. Furthermore, HCA and HDCA were shown as robust predictors of T2DM in two prospective longitudinal studies [104]. This discovery study established an associative relationship between HCA species and T2DM, suggesting that HCA species were crucial in glucose homeostasis [104].

### 4.2. Conjugated or Acylated BAs

Higher plasma/serum levels of glycine-conjugated BAs (glycocholic acid (GCA), GDCA, and glycochenodeoxycholic acid (GCDCA)) have been found to be positively correlated with T2DM risk in DPS and the Swedish meta-analysis study. These findings were further validated in the Nurses’ Health Study involving 448 American individuals with a median follow-up of 3.9 years [105]. The study in the 4C cohort consistently identified a relationship of higher T2DM risk with GCA and GCDCA. Taurine-conjugated BAs (taurocholic acid (TCA) and taurochenodeoxycholic acid (TCDCA)) and glycochenodeoxycholic acid (GCDCS) were also revealed to be associated with an elevated incidence of T2DM in the 4C cohort study. Moreover, multiple cross-sectional investigations have reported elevated levels of taurine-conjugated BAs (TCA, TDCA, and TCDCA) in T2DM [97,103,111] with positive correlations to both fasting and post-load glucose levels [111,112]. However, conflicting evidence exists regarding TCA’s role. In the Study of Latino Adolescents at Risk (SOLAR) with Hispanic adolescents, TCA correlated with elevated prediabetes risk, whereas the multi-ethnic MetaAir cohort linked it to reduced prediabetes risk [100]. Differences in gut microbiota composition between adolescents and adults may lead to variations in TCA production across these populations [113], and genetic differences among ethnic groups could further explain the observed heterogeneity in research findings. Regarding derivatives of UDCA, cross-sectional studies have shown a significant reduction in GUDCA levels in T2DM [94]. While numerous experimental studies have suggested that TUDCA can improve glucose homeostasis [114,115], supporting observational evidence remains limited. In fact, existing data indicate that elevated plasma levels of TUDCA are positively associated with an increased risk of T2DM [97]. Other BAs, such as GHCA, GHDCA, and THDCA, have been documented to diminish in T2DM [104,111], and glycine-conjugated GHCA and GHDCA were inversely associated with fasting or post-load blood glucose levels in serum and feces [104]. Notably, GHCA and GHDCA were also reported to be good predictors of T2DM in two longitudinal studies [104]. These results provided evidence that GHCA and GHDCA play critical roles in regulating glucose homeostasis and are protective against the development of T2DM in humans. 3-O-acyl-C has remained scarcely investigated in a large-scale study to date [27]. A case–control study involving 28 patients with T2DM and 18 healthy participants using the multiple reaction monitoring method based on LC-MS/MS to quantify human fecal 3-O-acyl-CA levels revealed a significant reduction in 3-O-acyl-CA in the T2DM [27].

### 4.3. Compare the Contributions of Conjugated and Unconjugated BAs to T2DM

Most unconjugated BAs (CA, CDCA, UDCA, and HCA) exhibit protective effects against T2DM. In contrast, the most abundant conjugated BAs in humans (including TCDCA, GCDCA, GCA, GDCA, and TDCA) [116] are positively associated with the risk of T2DM. However, it remains unclear whether the development of T2DM is primarily driven by the diminished protective effects of unconjugated BAs or by the enhanced diabetogenic effects of conjugated BAs. From a mechanistic perspective, unconjugated BAs, the most potent ligands for classical BA receptors that are primarily mechanisms for BAs in regulating glucose, have an advantage in receptor activation efficiency. However, the plasma levels of conjugated BAs are much higher than unconjugated BAs [116]. Therefore, the extent of their respective contributions warrants further exploration.

## 5. Bile Acid-Targeted Therapeutic Strategies for T2DM

### 5.1. Targeting the Enterohepatic Circulation of Bile Acids

Bile acid sequestrants (BASs) are a class of compounds that bind to BAs and prevent their reabsorption in the intestine, thereby promoting their excretion through feces [30]. This enhanced BA loss stimulates the hepatic conversion of cholesterol into BAs, thereby reducing circulating cholesterol levels [117]. Beyond lipid regulation, BASs have garnered attention for their potential to improve glycemic control in patients with T2DM. A meta-analysis comprising 17 randomized controlled trials demonstrated that BAS could reduce glycated HbA1c levels by 0.55% [118], but the exact mechanism underlying their glucose-lowering effects remains controversial. Early studies suggested that BASs might enhance glucose regulation by increasing BA concentrations in the ileum, thereby stimulating GLP-1 secretion [119]. However, this hypothesis has been challenged by recent findings. For instance, a clinical study observed that a single dose of colesevelam had no significant effect on postprandial GLP-1 response or glucose tolerance [120]. Moreover, sevelamer was found to reduce GLP-1 secretion in T2DM patients [121]. Interestingly, recent clinical research suggested that sevelamer improves insulin sensitivity and enhances β-cell responsiveness to glucose, with the latter effect being GLP-1 dose-dependent [122]. A recent mouse study identified FXR as a potential mediator of the glucose-lowering effects of colesevelam [123]. Therefore, the glucose-lowering mechanism of BAS requires additional investigation to clarify the precise pathway of its activity.

ASBT is responsible for the reabsorption of BAs in the ileum. Similar to BAS, ASBT inhibitors reduce intestinal BA absorption, decrease hepatic BAs, and promote cholesterol conversion into BAs [124]. ASBT inhibitors have been developed as a therapeutic approach for lowering low-density lipoprotein cholesterol [125,126]. Both human and animal studies have demonstrated that ASBT inhibitors can improve insulin sensitivity in diabetic patients [127]. This effect is partially attributed to the induction of GLP-1 secretion via the intestinal pathway [127].

### 5.2. Targeting Bile Acid Receptors

Obeticholic acid (OCA, INT-747) is a derivative of CDCA and a selective FXR agonist as a leading candidate in clinical trials for the treatment of non-alcoholic steatohepatitis (NASH) [128]. Preclinical studies have demonstrated that OCA can improve IR, a finding further supported by a clinical study in patients with T2DM and metabolic fatty liver disease [129,130]. However, a larger clinical trial reported that OCA might increase IR [131], indicating that its glucose-lowering effects require further validation.

6α-ethyl-23(S)-methyl-CA (EMCA or INT-777), a CA derivative, is TGR5 specific agonist. It exhibits multiple beneficial effects in vivo, including promoting GLP-1 secretion, increasing energy expenditure, reducing hepatic steatosis, and significantly mitigating weight gain and obesity risk [132]. Given the widespread distribution of TGR5 receptors across various tissues, the development of tissue-specific TGR5 agonists is considered a promising therapeutic approach [133]. In this regard, CA7S has been identified as a selective intestinal TGR5 activator, effectively minimizing the off-target effects of TGR5 agonists [65], making it a leading candidate for further development.

The semi-synthetic bile acid derivative INT-767 is the first dual agonist that can activate FXR and TGR5 efficiently and selectively [134]. Studies have shown that INT-767 exerts potent anti-inflammatory, antioxidant, and ER stress-relieving effects, along with restoring insulin sensitivity and notable renal protective effects in diabetic mice [135,136]. Notably, its renal effects appear to be non-redundant with other pathways, suggesting unique therapeutic potential [135]. However, whether dual activation provides superior glucose-lowering effects compared to single FXR or TGR5 agonists in humans remains to be confirmed through further clinical studies.

(E)-3α-Hydroxy-7-ethenyl-5β-cholan-24-oic acid (7-ELCA) is the first steroidal compound exhibiting dual activity as an FXR antagonist and a TGR5 agonist [137]. Studies have shown that 7-ELCA significantly stimulates GLP-1 secretion in intestinal enteroendocrine cells, thereby improving glucose metabolism [137]. As a potential therapeutic agent for T2DM, further research is needed to validate its efficacy and safety.

With growing interest in traditional Chinese medicine components, recent studies have shown that ginsenosides, the primary active constituents of ginseng, can regulate glucose homeostasis by inhibiting gluconeogenesis and enhancing glucose transport [138]. In mouse models, ginsenoside Ro has been demonstrated to activate TGR5, thereby stimulating GLP-1 secretion and enhancing energy expenditure, ultimately leading to improved IR [139]. Additionally, Notoginsenoside Ft1, derived from *Panax notoginseng*, exhibits dual activity as a TGR5 agonist and an FXR inhibitor [140]. By enhancing GLP-1 secretion and improving insulin sensitivity, it demonstrates potential antidiabetic effects [140].

### 5.3. Bile Acid Supplementation

UDCA and TUDCA have long been widely used for the treatment of liver diseases [107], and numerous animal studies and observational research suggest their positive role in maintaining glucose homeostasis [101,114,115]. A meta-analysis of seven clinical trials indicated that UDCA significantly reduces FBG, HbA1c, and insulin levels [109]. Furthermore, a recent phase II placebo-controlled randomized clinical trial involving 113 Chinese participants with a period of 12 weeks demonstrated that berberine ursodeoxycholate (HTD1801) significantly lowered HbA1c levels at week 12 and had a glycemic-lowering effect comparable to other antidiabetic drugs, indicating its potential as a therapeutic option for T2DM [141]. Notably, two phase III clinical trials assessing the efficacy and safety of HTD1801 in adult patients with T2DM who were inadequately controlled by diet and exercise or metformin treatment have been completed (NCT06350890 and NCT06353347). The trial results are pending publication and may substantiate HTD1801 as a prospective therapeutic alternative for T2DM. In contrast to BAS, which inhibits BA reabsorption, exogenously administered UDCA and TUDCA are effectively absorbed in the intestine, elevating BA levels in the human body to perform glucose-lowering effects. Increased research on TUDCA has revealed multiple glucose-regulating mechanisms, including inhibition of energy absorption, promotion of insulin secretion, and direct interaction with the insulin receptor, all of which underscore its therapeutic potential for T2DM [86,87,142,143]. Additionally, emerging evidence suggests that GUDCA may enhance thermogenesis, alleviate IR, and regulate TLCA levels, warranting further investigation into its therapeutic effects [94].

Beyond UDCA species, other BAs with potential benefits in glucose homeostasis remain underexplored. The gut-restricted TGR5 activation of CA7S [65], the dual TGR5 activation and FXR inhibition of HCA [67], and the FXR-inhibiting and insulin-sensitizing properties of 3-O-acylated bile acids [27] all deserve further high-quality preclinical and clinical studies to determine their therapeutic value for T2DM.

### 5.4. Challenges in Developing BA-Targeted Drugs

Despite the tremendous progress of BA-targeted drugs, numerous challenges persist. The first challenge is the translation of preclinical findings into clinical applications. Rodents and humans exhibit differences in the composition of the BA pool [144], the coding sequences of BA receptors [145], and the subsequent signaling pathways [146]. To address this, ongoing improvements in genetic and biochemical methodologies are essential to recreate the rodent BA pool and to humanize the coding sequences of BA receptors, hence enhancing the physiological relevance of rodent models for human research [147]. The second major challenge lies in the side effects of systemic BA receptors. The FXR agonist OCA has been reported to cause pruritus, a side effect potentially linked to the cytokine IL-31 [148]. Likewise, the TGR5 agonist INT-777 may cause excessive gallbladder distension and reduce susceptibility to cholecystokinin-induced contraction [149]. Notably, preclinical investigations have shown that gut-restricted BA receptors can significantly mitigate these adverse effects [147,150]. Similarly, innovative approaches like selective FXR modulators and novel non-steroidal scaffolds may reduce the incidence and severity of side effects [147]. These findings highlight the urgent need for drug development approaches, emphasizing tissue-specific targeting and molecular structural diversity.

## 6. Future Perspectives and Conclusions

Owing to the continuous innovation in metabolomics technologies, high-specificity and high-sensitivity MS techniques have enabled accurate detection and quantification of low-abundance BA molecules, driving rapid advancements in BA research. Numerous previously unrecognized BA modifications and structurally novel BAs have been identified, further expanding our understanding of BA diversity [20]. These technological breakthroughs allow systematic investigations into how distinct BAs influence T2DM, deepening our insights into the pathogenesis of T2DM and providing a theoretical basis for the development of new therapeutic strategies.

Nevertheless, challenges persist. While discrepancies in study design, population heterogeneity, or differential mechanistic contributions may partially explain conflicting results regarding the association between specific BAs (e.g., DCA, TCA) and T2DM risk, the implementation of more precise and standardized MS can minimize data variability and better characterize critical BA signatures. The potential role of newly discovered modified BAs, as identified by MS/MS spectra [20], in glucose metabolism, remains to be elucidated, necessitating advanced detection platforms with superior sensitivity and resolution. Moreover, MS serves a significant and adaptable function in assessing medication effects and can expedite the preclinical evaluation of therapeutic BA derivatives (e.g., HCA and CA7S). Hence, advancements in BA research for T2DM hinge fundamentally on methodological innovations.

In conclusion, distinct BAs have been demonstrated to exhibit varied roles in the pathogenesis and progression of T2DM. Ongoing advancements in metabolomics technologies will facilitate the detection of a more detailed and comprehensive BA profile, providing crucial insights and technical support for mechanistic understanding and developing targeted therapeutic strategies for T2DM.

## Figures and Tables

**Table 1:** Characteristics of studies investigating associations between bile acids and type 2 diabetes.

Study	Population	Study Design	Biological Sample	Methods	Tested Bile Acids	Key Findings	Ref.
China Cardiometabolic Disease and Cancer Cohort (4C) Study, Chinese	1707 matched case subject–control subject pairs with a median follow-up of 3.03 years	Nested case–control study	Fasting serum	Targeted, UPLC-MS/MS	23 BA species	Increased level in individuals with T2DM: GCDCA, GCA, GUDCA, GCDCAS, TCDCA, TCA, and TUDCA; Decreased level in individuals with T2DM: DCA, CA, and GCDCA-glucuronide Inversely associated with T2DM risk: CA, CDCA, and DCA; Positively associated with T2DM risk: GCA, TCA, GCDCA, TCDCA, and GCDCS	[97]
Uppsala Longitudinal Study of Adult Men (ULSAM), Swedish; Prospective Investigation of the Vasculature in Uppsala Seniors (PIVUS), Swedish; A case-cohort subset of the TwinGene study, Swedish; the Cooperative Health Research in the Region of Augsburg (KORA) S4 cohort, German	ULSAM: 1060 non-DM/78 T2DM, 21 years; PIVUS: 900 non-DM/70 T2DM, 5 years; TwinGene: 1508 non-DM/122 T2DM, 6 years; KORA S4: 767 non-DM/88 T2DM, 7 years.	Cohort, prospective, population-based	ULSAM: Plasma PIVUS, TwinGene and KORA: Serum	Non-targeted, LC-MS	5961 metabolic features (including BAs: not provided)	Associated with a high risk of prevalent T2DM: GDCA, GCA, DCA, and GCDCA.	[98]
Finnish Diabetes Prevention Study (DPS), Finland	96 T2DM, 5 years; 104 non-DM, 15 years	Nested case–control study	Fasting serum	Non-targeted, LC-MS	8607 metabolic features (including BAs: not provided)	Increased risk of T2DM: GCA, TCDCA, GCDCA, GDCA, DCA, and CA	[99]
Discovery Cohort: Latino Adolescents at Risk (SOLAR), Hispanic Replication Cohort: MetaAir cohort, mixed-ethnicity	SOLAR: 143 adolescents with overweight or obesity and without baseline prediabetes/38 prediabetes or T2DM, 1.2 years; MetaAir cohort: 56 young adults without baseline prediabetes/15 prediabetes or T2DM, 4.1 years	Cohort, prospective, population-based	Plasma (2h-OGTT)	Non-targeted, LC-HRMS	23,166 metabolic features (including BAs: not provided)	Discovery analysis: TCA was associated with an elevated risk of prediabetes; Replication analysis: TCA was associated with a reduced risk of prediabetes	[100]
Caregivers of the Birth to Twenty Plus cohort (BT20+), African	NGT-NGT: 28 individuals, 13 years NGT-IGT: 27 individuals, 13 years NGT-T2DM: 20 individuals, 13 years	Cohort, prospective, population-based	Serum	Non-targeted, GC-TOF/MS LC-TOF/MS	1076 putative metabolites with 252 identified metabolites (including 9 BAs)	At baseline: NGT-T2D group had lower levels of CDCA and UDCA At follow-up: NGT-T2D group had higher levels of DCA and GDCA	[101]
IGT Microbiota cohort, Swedish; Swedish Cardiopulmonary Bioimage Study (SCAPIS)-Gothenburg cohort, Swedish	IGT Microbiota cohort: 45 T2DM and 45 NGT; SCAPIS-Gothenburg cohort: 45 T2DM and 45 NGT	Cross-sectional	Plasma and fecal	Targeted, UPLC-MS/MS	28 BA species	Plasma levels increased in individuals with T2DM: DCA, isoDCA, 12-epiDCA, TDCA, GCA, GCDCA, GDCA, GHDCA, LCA, isoUDCA, and 12-oxoDCA; Both plasma and fecal DCA were positively associated with HOMA-IR, FBG, HbA1c, and insulin	[102]
Africa America Diabetes Mellitus study (AADM), Nigerians	Cross-sectional	Cross-sectional study	Plasma	Non-targeted, UPLC-MS/MS	1116 metabolites or compounds (including BAs: not provided)	Increased level in individuals with T2DM: GCA, TCA, DCA, GDCA, and TDCA	[103]
Volunteers, Chinese	28 T2DM/18 non-T2DM	Cross-sectional study	Fecal	Targeted, LC-QTOF	15 BA species (including 4 3-O-acyl-CAs)	Decreased level in individuals with T2DM: 3-O-acyl-CAs	[27]
Shanghai Obesity Study (SHOS), Chinese; Volunteers, Chinese; Shanghai Diabetes Study (SHDS), Chinese; Physical examination centers in Beijing, Chinese	SHOS: 1107 participants; Volunteers: 91 participants; SHDS: 132 participants, 10 years; Physical examination centers: 207 participants, 5 years	SHOS: Nested case–control study Volunteers: Cross-sectional study SHDS: longitudinal study Physical examination centers: longitudinal study	SHOS: Fasting serum Volunteers: Fasting serum and fecal SHDS: Fasting serum Physical examination centers: Fasting serum	Targeted, UPLC-MS	23 BA species (27 BAs species in physical examination centers)	Decreased level in individuals with T2DM: HCA, HDCA, GHCA, GHDCA; Inversely associated with fasting and post-load blood glucose levels: HCA, HDCA, GHCA, GHDCA; Decreased risk of T2DM: HCA, HDCA, GHCA, GHDCA	[104]
Nurses’ Health Study, American	224 matched case subject–control subject pairs with a median follow-up of 3.9 years	Nested case–control study	Blood	LC-MS	170 known metabolites (including 3 BAs)	Increased risk of T2DM: GCA, GDCA, GCDCA	[105]

**Table 2:** Observational associations and mechanisms of diverse bile acids in type 2 diabetes.

BAs	Key Findings from Observational Studies	Mechanism
Unconjugated BAs		
CA	Decreased risk of T2DM; Decreased level in T2DM	FXR (+): promote glycolysis, inhibit gluconeogenesis, enhance glycogen synthesis TGR5 (+): enhance GLP-1 secretion, promote glycolysis, inhibit gluconeogenesis, stimulate insulin release, inhibit glucagon secretion, enhance energy expenditure
CDCA	Decreased risk of T2DM;	FXR (+): promote glycolysis, inhibit gluconeogenesis, enhance glycogen synthesis; TGR5 (+): enhance GLP-1 secretion, promote glycolysis, inhibit gluconeogenesis, stimulate insulin release, inhibit glucagon secretion, enhance energy expenditure; Non-receptor-mediated: bind and stabilize NAPE-PLD to inhibit gluconeogenesis, enhance glycogen synthesis and GLP-1 secretion, enhance FOXO1 activity to improve insulin sensitivity
HCA	Decreased risk of T2DM; Inversely associated with HOMA-IR, FBG, and post-load blood glucose levels	FXR (−): enhance GLP-1 secretion; TGR5 (+): enhance GLP-1 secretion
DCA *	Increased/Decreased risk of T2DM; Increased/Decreased level in T2DM; Positively associated with HOMA-IR, FBG, HbA1c, and insulin	FXR (+): promote glycolysis, inhibit gluconeogenesis, enhance glycogen synthesis TGR5 (+): enhance GLP-1 secretion, promote glycolysis, inhibit gluconeogenesis, stimulate insulin release, inhibit glucagon secretion, enhance energy expenditure; Non-receptor-mediated: Bind and stabilize NAPE-PLD; affect membranes; upregulate the expression of *ATGL*, *HSL*, and *UCP1*
LCA	Increased level in T2DM	FXR (+): promote glycolysis, inhibit gluconeogenesis, enhance glycogen synthesis; TGR5 (+): enhance GLP-1 secretion, promote glycolysis, inhibit gluconeogenesis, stimulate insulin release, inhibit glucagon secretion, enhance energy expenditure; VDR(+): maintain immunity homeostasis, enhance GLP-1 and insulin secretion PXR(+) CAR(+): inhibit CYP7A1
UDCA	Decreased risk of T2DM	FXR (−): improve insulin resistance, inhibit glucose absorption, enhance GLP-1 secretion; TGR5 (+): enhance GLP-1 secretion, promote glycolysis, inhibit gluconeogenesis, stimulate insulin release, inhibit glucagon secretion, enhance energy expenditure
HDCA	Decreased risk of T2DM; Inversely associated with fasting and post-load blood glucose	FXR (−): improve insulin resistance, inhibit glucose absorption, enhance GLP-1 secretion; TGR5 (+): enhance GLP-1 secretion, promote glycolysis, inhibit gluconeogenesis, stimulate insulin release, inhibit glucagon secretion, enhance energy expenditure
Conjugated BAs		
GCA	Increased risk of T2DM; Increased level in T2DM;	TGR5 (+): enhance GLP-1 secretion, promote glycolysis, inhibit gluconeogenesis, stimulate insulin release, inhibit glucagon secretion, enhance energy expenditure
TCA *	Increased/Decreased risk of T2DM; Increased level in T2DM	TGR5 (+): enhance GLP-1 secretion, promote glycolysis, inhibit gluconeogenesis, stimulate insulin release, inhibit glucagon secretion, enhance energy expenditure
GCDCA	Increased risk of T2DM; Increased level of T2DM;	TGR5 (+): enhance GLP-1 secretion, promote glycolysis, inhibit gluconeogenesis, stimulate insulin release, inhibit glucagon secretion, enhance energy expenditure
TCDCA	Increased risk of T2DM	FXR (+): promote glycolysis, inhibit gluconeogenesis, enhance glycogen synthesis, aggravate insulin resistance
GHCA	Decreased risk of T2DM; Decreased level in T2DM; Inversely associated with fasting and post-load blood glucose	FXR (−): enhance GLP-1 secretion; TGR5 (+): enhance GLP-1 secretion
THCA	Not reported	FXR (−): enhance GLP-1 secretion; TGR5 (+): enhance GLP-1 secretion
GDCA	Increased risk of T2DM	TGR5 (+): enhance GLP-1 secretion, promote glycolysis, inhibit gluconeogenesis, stimulate insulin release, inhibit glucagon secretion, enhance energy expenditure; Non-receptor-mediated: inhibits insulin clearance to aggravate insulin resistance
TDCA	Increased level in T2DM; Positively associated with FGB/HOMA-IR	TGR5 (+): enhance GLP-1 secretion, promote glycolysis, inhibit gluconeogenesis, stimulate insulin release, inhibit glucagon secretion, enhance energy expenditure; Non-receptor-mediated: downregulate the expression of *CAR 1* and upregulate the expression of UCP-1 to enhance energy expenditure, inhibit insulin clearance to aggravate insulin resistance
GLCA	Not reported	TGR5 (+): enhance GLP-1 secretion, promote glycolysis, inhibit gluconeogenesis, stimulate insulin release, inhibit glucagon secretion, enhance energy expenditure
TLCA	Not reported	TGR5 (+): enhance GLP-1 secretion, promote glycolysis, inhibit gluconeogenesis, stimulate insulin release, inhibit glucagon secretion, enhance energy expenditure
GUDCA	Decreased level in T2DM	FXR (−): improve insulin resistance, inhibit glucose absorption, enhance GLP-1 secretion; TGR5 (+): enhance GLP-1 secretion, promote glycolysis, inhibit gluconeogenesis, stimulate insulin release, inhibit glucagon secretion, enhance energy expenditure
TUDCA	Increased risk of T2DM	FXR (−): improve insulin resistance, inhibit glucose absorption, enhance GLP-1 secretion; TGR5 (+): enhance GLP-1 secretion, promote glycolysis, inhibit gluconeogenesis, stimulate insulin release, inhibit glucagon secretion, enhance energy expenditure; Non-receptor-mediated: improve pancreatic islet function and enhance insulin secretion by alleviating ER stress, blocking SUR1, binding with insulin receptors, and activating the cAMP/PKA/CREB signaling pathway
GHDCA	Decreased risk of T2DM; Decreased level in T2DM; Inversely associated with fasting and post-load blood glucose	FXR (−): enhance GLP-1 secretion; TGR5 (+): enhance GLP-1 secretion
THDCA	Decreased level in T2DM;	FXR (−): enhance GLP-1 secretion; TGR5 (+): enhance GLP-1 secretion
3-O-acyl-CA	Decreased level in T2DM	FXR (−): enhance energy metabolism and insulin sensitivity Non-receptor-mediated: promote microbial balance
CA7S	Not reported	TGR5(+): enhance GLP-1 secretion

(+), agonists; (−), inhibitors; * conflicting findings from the observational studies.

## Data Availability

Not applicable.

## Abbreviations

The following abbreviations are used in this manuscript: Type 2 diabetes mellitus (T2DM); insulin resistance (IR); bile acid (BA); farnesoid X receptor (FXR); Takeda G protein-coupled receptor 5 (TGR5); glucagon-like peptide-1 (GLP-1); liquid chromatograph mass spectrometer (LC-MS); tandem mass spectrometry (MS/MS); cholic acid (CA); chenodeoxycholic acid (CDCA); cholesterol 7α-hydroxylase (CYP7A1); sterol 12α-hydroxylase (CYP8B1); sterol 27-hydroxylase (CYP27A1); oxysterol 7α-hydroxylase (CYP7B1); hyocholic acid (HCA); CoA synthetase; bile acid–CoA: amino acid N-acyltransferase (BAAT); bile salt export pump (BSEP); bile salt hydrolase (BSH); deoxycholic acid (DCA); lithocholic acid (LCA); ursodeoxycholic acid (UDCA); 3-acetyl cholic acid (Ac-CA); 3-propionyl cholic acid (Prp-CA); 3-butyryl cholic acid (Buty-CA); 3-valeryl cholic acid (Val-CA); bile acid transporter (ASBT); organic solute transporter alpha and beta (OST alpha/bet); ileum bile acid binding protein (IBABP); sodium-dependent taurocholate cotransporting polypeptide (NTCP); small heterodimer partner (SHP); glucose-6-phosphatase (G6Pase); phosphoenolpyruvate carboxylase kinase (PEPCK); carbohydrate response element-binding protein (ChREBP); fibroblast growth factor 19 (FGF19); endoplasmic reticulum (ER); glycoursodeoxycholic acid (GUDCA), tauroursodeoxycholic acid (TUDCA); hyodeoxycholic acid (HDCA), glycohyocholic acid (GHCA), glycohyodeoxycholic acid (GHDCA), taurohyocholic acid (THCA), taurohyodeoxycholic acid (THDCA); taurochenodeoxycholic acid (TCDCA); high-fat diet (HFD); dorsal vagal complex (DVC); brown adipose tissue (BAT); peptide tyrosine tyrosine (PYY); thyroxine (T4); triiodothyronine (T3); cholic acid-7-sulfate (CA7S); vitamin D receptor (VDR); sulfotransferase (SULT2A); pregnane X receptor (PXR); constitutive androstane receptor (CAR); N-acyl phosphatidylethanolamine phospholipase D (NAPE-PLD); oleoylethanolamide (OEA); sulfonylurea receptor 1 (SUR1); glutamate dehydrogenase (GDH); factor forkhead box O1 (FOXO1); uncoupling protein 1 (UCP-1); carbonic anhydrase 1 (CAR1); glycodeoxycholic acid (GDCA); taurodeoxycholic acid (TDCA); taurolithocholic acid (TLCA);carbonic anhydrase 1 (CAR1); China Cardiometabolic Disease and Cancer Cohort (4C cohort); Diabetes Prevention Study (DPS); normal glucose regulation (NGR); impaired fasting glucose (IFG); fasting blood glucose (FBG); glycated hemoglobin (HbA1c); glycocholic acid (GCA); glycochenodeoxycholic acid (GCDCA); taurocholic acid (TCA); glycochenodeoxycholic acid (GCDCS); Study of Latino Adolescents at Risk (SOLAR); bile acid sequestrants (BAS); obeticholic acid (OCA); (E)-3α-Hydroxy-7-ethenyl-5β-cholan-24-oic acid (7-ELCA).
